# Diagnostic Accuracy of Fine Needle Aspiration Cytology in Lesions of Oral Cavity and Salivary Glands: A Clinico-Pathological Study

**DOI:** 10.2174/1745017901814010782

**Published:** 2018-09-28

**Authors:** Shubhangi Shalley, Nasib Chand, Amit Aggarwal, Laxmi Narayan Garg, Varuni Yadav, Aashit Yadav

**Affiliations:** 1 Department of Pathology, Maharishi Markandeshwar Institute of Medical Sciences and Research, Mullana, Ambala, India; 2 Department of Oral Medicine and Radiology, Maharishi Markandeshwar College of Dental Science and Research, Mullana, Ambala, India; 3 Department of Otolaryngeology, Maharishi Markandeshwar Institute of Medical Sciences and Research, Mullana, Ambala, India

**Keywords:** FNAC, Oral lesions, Salivary gland tumors, Cytological diagnosis, Accuracy, Hard palate

## Abstract

**Objective:**

Fine Needle Aspiration Cytology (FNAC) is a rapid, reliable and safe diagnostic tool used for various lesions of the oral cavity and salivary glands. The present study was undertaken to categorize the cytomorphology of the oral cavity and salivary gland lesions on FNAC and to assess the accuracy of FNAC in arriving at a diagnosis.

**Materials and Methods:**

A prospective study on oral cavity swellings and salivary gland aspirates was done during a 2 year period from August 2015 to July 2017 in which a total of 70 FNAC’s were performed. There were 12 aspirates obtained from oral cavity swellings and 58 aspirates were obtained from salivary glands. Histopathological evaluation of 65 lesions was done and was considered as gold standard. Only the lesions undergoing histopathological confirmation were included in the study. The sensitivity, specificity, diagnostic accuracy and clinical utility index were evaluated for accuracy of FNAC.

**Results:**

Hard palate (33.33%) was the predominantly aspirated site in the oral cavity. Parotid gland was the predominant gland aspirated (60.32%) among the involved salivary glands. Non-neoplastic lesions constituted 18.47% cases whereas neoplastic lesions were 81.53% (60.00% benign and 21.53% malignant). Pleomorphic adenoma (28.65%) was the most common benign lesion in the oral cavity involving hard palate and as salivary gland neoplasm (70.54%). Squamous cell carcinoma (60%) was the most common malignant lesion of oral cavity involving the tongue and buccal mucosa and adenoid cystic carcinoma (44.45%) was the commonest malignancy in salivary gland malignant neoplasms. The overall sensitivity, specificity and accuracy of FNAC in the present study were 89.5%, 100% and 85% respectively.

**Conclusion:**

FNAC is a safe, cost-effective and reliable technique effective in diagnosing the spectrum of different lesions in the oral and maxillofacial region.

## 1 BACKGROUND

The oral cavity is affected by a wide variety of pathologic lesions that need an accurate diagnosis for guiding further management [1, 2]. Oral and oropharyngeal mass lesions are commonly diagnosed by biopsy [3]. Traditional biopsy techniques in the oral cavity may require anesthesia and may have diagnostic difficulties especially for trans-mucosal lesions [3].

The need for an accurate, timely and well- structured pathology report has become increasingly important in this age, considering a society that is both erudite and critical. Fine Needle Aspiration Cytology (FNAC) is shown to be a safe and reliable method that overcomes these problems by providing a minimally invasive means to rapid diagnosis of intraoral lesions, and if necessary, a re-aspiration can be done immediately [2, 4]. This technique has rarely been used to diagnose oral and pharyngeal lesions but has become a diagnostic test of choice for salivary gland lesions [2-4]. First described by Kun in 1847, the modern method of FNAC was introduced by Martin and Ellis in 1930; fine-needle aspiration can be performed either with or without imagistic assistance (ultrasound)., It provides prompt information about the nature of the assessed lesion. The technique has very few contraindications and risks, and it is suitable for use in an ambulatory setting. FNAC differentiates nonneoplastic lesions from neoplastic lesions thus eliminating the need for surgical intervention in these lesions which can be treated conservatively [4].

The global annual incidence of salivary gland tumors varies from 0.4-13.5 cases per 100,000 population [5]. FNAC serves to determine the nature of the lesion which can be divided into inflammatory, benign and malignant and in some cases, the specific diagnosis is given [6, 7]. Malignant salivary gland neoplasms account for more than 0.5% of all malignancies and approximately 3% to 5% of all head and neck cancers [8]. Pleomorphic adenoma of salivary glands is the most frequently encountered benign tumor [9, 10] and squamous cell carcinoma is the most common malignancy reported in the oral cavity [11, 12].

Adequate cellularity of the smears and proper sampling of lesions is the pre-requisites for an accurate diagnosis [10]. The high sensitivity, specificity and diagnostic accuracy of FNAC confirm its vital role along with clinical and radiological findings to provide the best possible initial evaluation, which in turn guide the treatment options [13]. The aim of the present study was to study the cytological spectrum of lesions of the oral cavity and salivary glands, to evaluate the role of fine needle aspiration cytology as a diagnostic investigation for lesions of the oral cavity and salivary glands and to correlate cytological diagnosis with histopathological diagnosis.

## 2 MATERIALS AND METHODS

The prospective study was done on FNAC slides obtained from 70 cases of oral cavity swellings and salivary glands lesions analyzed by light microscopy (Nikon clinical microscope) in the cytology section of pathology department from August 2015 to July 2017 and 65 cases underwent histopathological assessment, which were included in the study. All the swelling cases of Oral Cavity and salivary glands reporting to the Department of Oral Medicine and Radiology were clinically diagnosed by a trained Oral Physician and the cases were referred to the Department of General Pathology for confirmation by FNAC and biopsy. The sample size was selected fulfilling the statistically validated criteria. A written consent from all the included patients was taken. The criteria for inclusion was the patients presenting with superficial, palpable and nodular lesions of the oral cavity and salivary glands, having size more than 1 cm. Nodular and superficial lesions like: pleomorphic adenoma, carcinoma and salivary gland tumors which were visible clinically were included. All the swellings were examined clinically on inspection and palpation along with approximate size more than 1 cm and the absence of any contraindications for performing fine-needle aspiration like highly vascular lesions, patients with bleeding disorders or patients on anticoagulant therapy, uncooperative or excessively apprehensive patient [14]. All the superficial flat and deep-seated lesions were excluded like macules, papules, dermoid cyst, subcutaneous neurofibroma, port-wine lesions, verrucous keratosis *etc*. All the cases were clinically diagnosed by the maxillofacial diagnostician, FNAC was carried out by a general surgeon and all slides were diagnosed by the pathologist. The study was approved by the institutional ethical committee vide *letter no. MM 15/639.*

For FNAC, the following technique was used [15]: The skin was cleaned with spirit. A standard procedure using 22-23 gauge needles connected to a 10 c.c. plastic syringe was used for all FNAC techniques. The area to be aspirated was immobilized with thumb and index finger. The needle was placed against the skin at the determined puncture site and inserted into the mass, with the single quick motion without negative pressure in the syringe. Once the needle was in the mass, plunger of the syringe was retracted to create negative pressure in the syringe and lumen of the needle. This drew the material into the needle. The needle was moved back and forth 3-4 times and directed into different areas of the mass. The plunger was released and the needle was withdrawn from the lesion and pressure was applied on the puncture with spirit soaked cotton. The aspirate was expressed on the slides and smears were made by applying a gentle pressure with another slide. May-Grunwald Giesma (MGG) staining, Papanicolau (PAP) staining and Hematoxylin and Eosin (H&E) staining was done using standard guidelines [16, 17] for the cytological diagnosis. For PAP and H&E staining slides were immediately fixed in Carnoy’s fixative or 95% ethanol and for MGG staining slides were air dried. The slides were reviewed 3 times at separate intervals by the same pathologist for intra-observer differences which were none. To corroborate the findings, histopathological diagnosis was also done and considered as gold standard. Special stains and markers were used wherever required.

To evaluate the accuracy of FNAC in the diagnosis of lesions of the oral cavity and salivary glands; sensitivity, specificity, positive predictive value and negative predictive value was evaluated, with *SPSS* 15.0 programme. The Clinical utility index was also calculated with 95% confidence level [18].

## 3 RESULTS

The age of the patients with lesions of the oral cavity and salivary glands ranged from 13 to 85 years with a mean age of 49 years. The most affected age group was 41-50 years (n=16). Fifteen patients belonged to 31-40 year age group, twelve patients were less than 30 years of age and 6 patients were in 10-20 year age group. Eight patients were in 51-60 age group and 8 patients were in the age group above sixty (Table **1**). Among the cases studied, 42 cases were males (64.61%) and 23 cases were females (35.39%). The male to female ratio was 1.8:1.

Hard palate (33.33%) was the predominantly aspirated site which was followed by tongue and buccal mucosa (25% each). Mandible was involved in 16.67% cases. Parotid gland constituted the commonest site involved in salivary gland lesions (60.32%), followed by the submandibular gland (32.77%) and sublingual gland (6.91%) (Table **2**).

Pleomorphic adenoma (28.65%) was the most common benign lesion in the oral cavity involving hard palate and as salivary gland neoplasm (70.54%). Squamous cell carcinoma (60%) was the most common malignant lesion of oral cavity involving the tongue and buccal mucosa, and Adenoid cystic carcinoma (44.45%) was the most commonly found malignancy among salivary gland malignant neoplasms followed by mucoepidermoid carcinoma (22.2%) (Table **3**). Warthin’s Tumor was present in 26.5% cases. A single case of schwannoma was seen involving the submandibular gland in a 38 year old male (Table **3**).

The overall sensitivity, specificity and accuracy of FNAC in the present study were 89.5%, 100% and 85% respectively (value missing). The positive predictive value and negative predictive value were 100% and 50.0% respectively with excellent (1.029) clinical utility index.

## 4 DISCUSSION

Biopsy is the commonly used diagnostic procedure for intraoral hard and soft tissue lesions and histopathology is considered the gold standard for diagnosis. However, FNAC is an acceptable alternative procedure with an excellent diagnostic accuracy. FNAC is the method of choice for tumors located in the salivary glands [4, 19]. Even in salivary gland tumors that present with many diagnostic difficulties, accuracy of 90% has been reported [19]. FNAC is considered safe, simple, rapid, repeatable and relatively cheap [2-4]. They leave no scars and there is no risk of seeding tumors along the needle tract [19].

The age of the patients varied from 13 to 85 years with a maximum number of patients falling in the age group of 30 to 50 years. Issac *et al*. [20], and Kakoty *et al*. [21], reported similar findings. According to Rutt AL *et al*. [22], it is essential for physicians to detect salivary gland neoplasms promptly and to evaluate them thoroughly when they are found in young age. FNAC has an edge over biopsy as cytological diagnosis can be made within 24 hours whereas histopathology may take 5-7 days [15]. For malignant lesions, these intervening days are important as the treatment may be started at the earliest possible time following diagnosis to reduce morbidity and mortality.

Cytological results of the oral cavity were categorized into benign and malignant lesions, with a predominance of benign lesions over malignant lesions. FNAC showed the highest accuracy (90%) in differentiating benign from malignant tumors which was the same in our findings. Highest concordance of FNAC with histopathological diagnosis was in cases of benign lesions. Core needle biopsy (97%) and open biopsy (100%) are more accurate but are invasive. Among the benign lesions, pleomorphic adenoma involving the hard palate was the most common lesion. Atanda *et al*. [23], also reported pleomorphic adenoma as the most frequent benign lesion (47.85%). Cytologically, smear shows scattered and irregular groups of epithelial cells with interspersed chondromyxoid stromal fragments and background population of myoepithelial cells.

A single case of central giant cell granuloma was seen in a 24-year old male involving the mandible with the previous history of tooth extraction. Cytologically, smears show a dual population of osteoclast-like giant cells and spindle-shaped mononuclear stromal cells. In one case reported as ameloblastoma involving the hard palate in a 50 year old female, smears revealed a cohesive cluster of basaloid epithelial cells with peripheral palisading and polygonal squamous cells in a proteinaceous background. One case each of acute inflammatory pathology, mucocele and lymphoepithelial cyst was also seen.

**Fig. (1):**
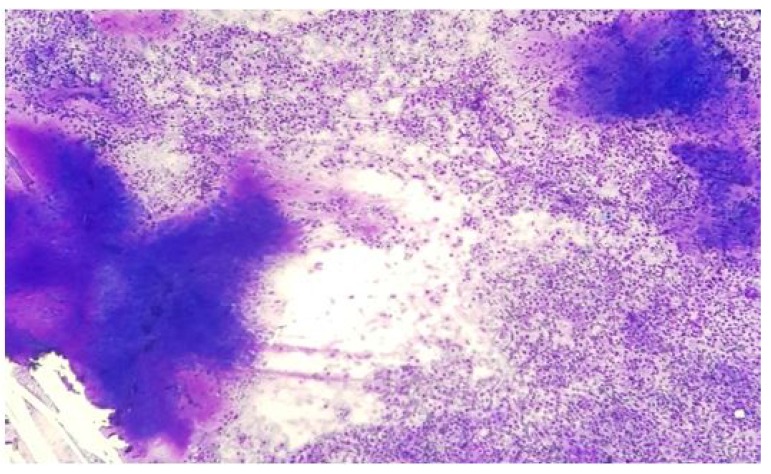
Pleomorphic adenoma – cytology: Smear showing singly scattered and irregular groups of epithelial cells with interspersed chondromyxoid stromal fragments and background population of myoepithelial cells (MGG, X100).

In the salivary gland lesion cases, inflammatory lesions were the most common non-neoplastic lesions. Sialadenitis constituted the majority of non-neoplastic lesions, comprising chronic sialadenitis and granulomatous sialadenitis. Pleomorphic adenoma was the most common benign salivary gland neoplasm (Fig. **1**) followed by Warthin’s Tumor. Stewart *et al*. [3], and Naz *et al*. [4], also documented similar findings with PA constituting the maximum cases followed by Warthin’s Tumor. A case of mucoepidermoid carcinoma was misdiagnosed on FNAC as pleomorphic adenoma. One of the major challenges in salivary gland cytology is the accurate separation of biphasic basaloid neoplasms such as pleomorphic adenoma, basal cell neoplasms (adenoma and adenocarcinoma), epithelial-myoepithelial carcinoma, and adenoid cystic carcinoma [19]. A single case of schwannoma was seen involving submandibular gland in a 38 year old male. Ersoz *et al*. [24], reported 2 cases of schwannoma in their study. The cytological smear revealed small fascicles, singly scattered spindle-shaped tumor cells, elongated wavy nuclei with pointed ends, bland nuclear chromatin with mild pleomorphism embedded in a fibrillary eosinophilic matrix representing Antoni B areas.

**Fig. (2):**
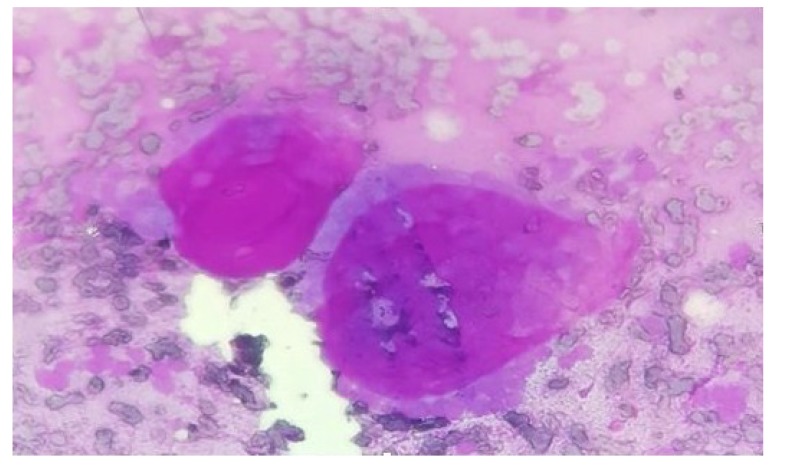
Adenoid cystic carcinoma – cytology: Small round cells with scant cytoplasm arranged around pinkish hyaline globules (MGG, X400).

**Fig. (3):**
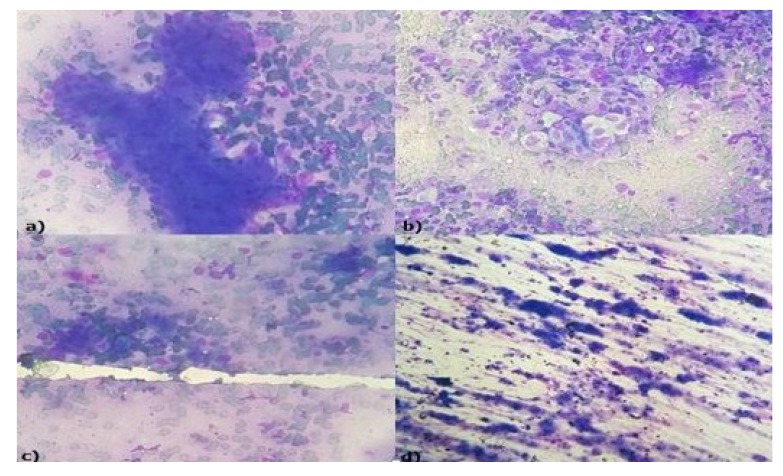
**(a)** Squamous cell carcinoma - cytology: Clusters of malignant squamous cells (PAP, X100). **(b)** Orangeophilic malignant squamous cells with hyperchromatic nuclei (PAP, X400).

Adenoid cystic carcinoma (Fig. **2**) was the predominant malignancy in salivary gland neoplasms followed by mucoepidermoid carcinoma (Fig. **3**). Cytological section showed large globules of the extracellular matrix, partially surrounded by basaloid tumor cells. Similar findings were seen in studies conducted by Todase *et al*. [25], whereas in a study conducted by Sushma *et al*. [26], mucoepidermoid carcinoma was the most frequent malignant neoplasm. One case each of polymorphous low-grade adenocarcinoma, salivary duct carcinoma and basal cell adenocarcinoma was seen arising from parotid gland.

**Fig. (4):**
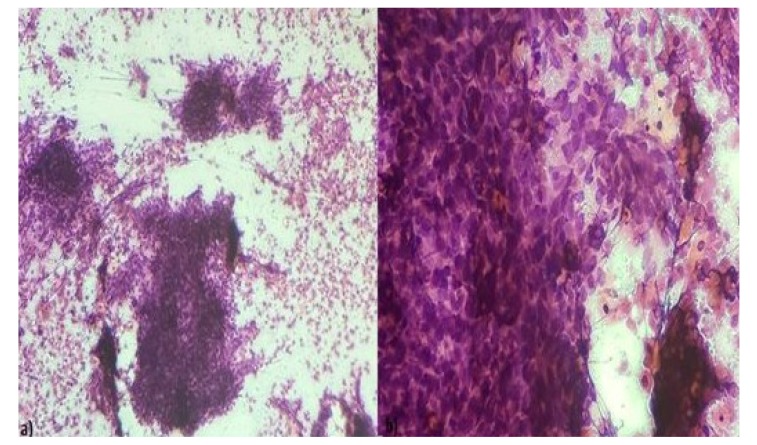
**(a)** Mucoepidermoid carcinoma – cytology: Intermediate cells in cluster (MGG, X400). **(b)** Mucin producing cells with abundant vacuolated cytoplasm (MGG, X400). **(c)** Squamous cells (MGG, X400). **(d)** Dirty mucoid background (MGG, X100).

Squamous cell carcinoma (Fig. **4**) was the most common malignant lesion of the oral cavity constituting 60% of malignancies involving the tongue and buccal mucosa. The cytological smear revealed sheets, clusters, and scattered malignant squamous cells with anaplasia, hyperchromatic nuclei, and dense cytoplasm. In the study conducted by Sakarwal *et al*. [27], squamous cell carcinoma was the most common malignancy constituting 79.31% of malignant lesions of the oral cavity. Hafez *et al*. [15], also reported squamous cell carcinoma as the most commonly found malignancy. One case of Polymorphous low-grade adenocarcinoma was seen in an 80 year old female arising from the hard palate and another case of poorly differentiated carcinoma of the mandible in a 65 year old male. The results of the present study were also compared with a study done by Alina Iacob *et al*., of Romania, who performed a prospective cross-sectional study on 58 patients in which Cytology had 76.5% specificity and 78.1% sensitivity for identifying malignant lateral cervical lesions [28]. The high sensitivity (89.5%), specificity (100%) and accuracy (85%) of FNAC in the results have also been found by many authors [13, 29] especially for salivary gland lesions resulting in an acceptable view that FNAC can reliably be used as a preliminary diagnostic method in the majority of oral and salivary lesions with a clinical utility index of 1.029.

The limitation of the current study was limited number of oral lesions which were less as compared to salivary gland lesions but when considered in the maxillofacial area the studied sample size was adequate. The study could have included cysts and periapical abscess affecting the oral cavity which could have been diagnosed fairly accurately with FNAC. However, inflammatory conditions of the oral cavity are readily diagnosed clinically and radiographically; the main area of misdiagnosis comes with benign and malignant lesions which require fast and accurate diagnosis for the treatment to be commenced immediately. One malignant case was misdiagnosed on FNAC which could lead to complications. Proper sampling of lesions and adequate cellularity of smears are pre-requisites for an accurate diagnosis. Further studies are necessary in which more emphasis can be laid on oral lesions.

## CONCLUSION

It is concluded that FNAC can be widely used in the diagnosis of the oral cavity and salivary gland lesions with high accuracy, rapid results and excellent clinical utility index. It helps in an early diagnosis and in distinguishing benign from malignant lesions. Thus FNAC is a safe, reliable and yet economically effective procedure.

What is already known:

- FNAC is considered safe, simple, rapid, repeatable and relatively cheap.
- FNAC is the method of choice for tumors located in the salivary glands.

What this study adds:

- Diagnostic accuracy of FNAC in oral lesions.
- The variety of the oral and salivary gland lesions.

## Figures and Tables

**Table 1:** Age distribution in lesions of oral cavity and salivary glands.

ORAL CAVITY			SALIVARY GLANDS		
**Age Group (years)**	**No. of Cases**	**Percentage**	**Age Group (years)**	**No. of Cases**	**Percentage**
–	–	–	10-20	6	10.32%
Less than 30	2	16.67%	21-30	10	17.23%
31-40	1	8.33%	31-40	14	24.17%
41-50	4	33.33%	41-50	12	20.64%
51-60	1	8.33%	51-60	7	12.1%
61-70	1	16.67%	61-70	5	12.1%
More than 70	1	16.67%	More than 70	1	3.44%
Total	10	100%	Total	55	100%

**Table 2:** Site distribution in lesions of oral cavity and salivary glands.

ORAL CAVITY			SALIVARY GLANDS		
**Site**	**No. of Cases**	**Percentage**	**Site**	**No. of Cases**	**Percentage**
Hard Palate	4	33.33%	Parotid	35	60.32%
Tongue	2	25%	Submandibular	17	32.77%
Buccal mucosa	2	25%	Sublingual	3	6.91%
Mandible	2	16.67%	-	-	-
Total	10	100%	Total	55	100%

**Table 3:** Number of cases based on cytological and pathological diagnosis in the oral cavity and salivary glands (Total cases – 65).

**Types of Case**	**Cytological** **Diagnosis**	**Histopathology (Concordant)**	**Discordant (Benign)**	**Discordant (Malignant)**
**Non-Neoplastic Lesions**		-	-	-
Chronic Sialadenitis	8 (12.3%)	5	3	-
Granulomatous Sialadenitis	2 (3.07%)	2	-	-
Mucocele	2 (3.07%)	2	-	-
**Total**	**12 (18.47%)**	**9**	**-**	**-**
**Benign Lesions**		-	**-**	**-**
Central giant cell granuloma	1 (1.54%)	1	-	-
Lymphoepithelial cyst	1 (1.54%)	1	-	-
Ameloblastoma	1 (1.54%)	1	-	-
Pleomorphic adenoma	26 (40.0%)	24	1	1
Warthin’s Tumor	9 (13.84%)	9	-	-
Schwannoma	1 (1.54%)	1	-	-
**Total**	**39 (60.0%)**	**37**	**-**	**-**
**Malignant Lesions**		**-**	**-**	**-**
Squamous cell carcinoma	4 (6.15%)	3	-	1
Adenoid cystic carcinoma	4 (6.15%)	3	1	-
Mucoepidermoid carcinoma	2 (3.07%)	1	1	-
Polymorphous low grade Adenocarcinoma	2 (3.07%)	2	-	-
Salivary duct carcinoma	1 (1.54%)	1	-	-
Basal cell adenocarcinoma	1 (1.54%)	1	-	-
**Total**	**14 (21.53%)**	**11**	**-**	**-**
**Total of all lesions**	**65 (100%)**	**57**	**6**	**2**
